# Supplementary material for: Association between COVID-19 Vaccine Side Effects and Body Mass Index in Spain
Source: Vaccines (Basel). 2021 Nov 15;9(11):1321. doi: 10.3390/vaccines9111321 (PMC8623604; doi:10.3390/vaccines9111321)
Supplement: Supplementary file 1 [file vaccines-09-01321-s001.zip › vaccines-1415428-supplementary.pdf]

SUPPLEMENTARY MATERIAL

Table S1. Other sociodemographic information of the sample characteristics (Total sample: N=2,136; vaccinated with at least one dose: n=1,189 and with required doses: n=590).

**N=2,136**	**N**	**%**
**Born in Spain**		
Yes	1,977	92.6
No	136	6.4
**Occupation**		
Healthcare Professional	664	31.1
Non-healthcare Professional	986	46.2
Retired	157	7.4
Unemployed or Student	329	15.4
**Vaccination against COVID-19**		
No, for medical reasons	13	0.6
No, because I was pregnant	18	0.8
No, because they did not offer me the vaccine yet	825	38.6
No, because I just had COVID-19	59	2.8
No, because I refused to	32	1.5
Yes, but I only got one dose (and I need two doses)	599	28.0
Yes, and I got all doses	590	27.6
**Did you get infected with COVID-19?**		
No	1642	76.5
Yes, and I had a positive PCR	232	10.8
Yes, and I had a positive test different than PCR	97	4.5
Yes, I think I had it, but I did get a test, or the test was negative	165	7.7

Missing	10	0.5
**N= 1,189 vaccinated with at least one dose**	**N**	**%**
**Possibility of being vaccinated after knowing side effects**		
Yes, of course	1057	88.9
Yes, but I would think about it more	34	2.9
I will have doubts	32	2.7
No, side effects that I had do not make up for it	11	0.9
Missing	55	4.6
**Side effects first dose**		
**Age (in years)**		
18-25	210	17.1
26-35	96	8.1
36-45	191	16.1
46-55	174	14.1
56-65	297	25.0
>65	221	18.6
**Gender**		
Male	338	28.4
Female	851	71.6
**Side effects of second dose or required dose* (N=590)**		
**Age (in years)**		
18-25	27	4.6
26-35	69	11.7
36-45	106	18.0
46-55	116	19.7
56-65	145	24.6
>65	127	21.5
**Gender**		
Male	160	27.1

Female	430	72.9
**Body Mass Index** (Mean and Standard deviation)		
	24.69	4.35
**Fever<38º**		
Yes	82	13.0
No	508	86.1
**Fever≥38º**		
Yes	40	6.8
No	540	93.2
**Myalgia (muscle pain)**		
Yes	145	24.6
No	445	75.4
**Arm soreness, redness, swelling**		
Yes	194	32.9
No	393	67.1
**Nausea**		
Yes	36	6.1
No	554	93.9
**Vomiting**		
Yes	11	1.9
No	579	98.1
**Red, itchy, swollen, or painful rash**		
Yes	7	1.2
No	583	98.8
**Headache**		
Yes	135	22.9
No	455	77.1
**Diarrhea**		
Yes	11	1.9

No	579	98.1
**Loss of appetite**		
Yes	19	3.2
No	571	96.8
**Sweating**		
Yes	19	3.2
No	571	96.8
**Chills**		
Yes	82	13.2
No	512	86.8
**Enlarged lymph nodes**		
Yes	21	3.6
No	569	96.4
**Altered menstrual cycle**		
Yes	3	0.5
No	587	99.5
**Tiredness, sleepiness, dizziness**		
Yes	62	10.5
No	528	89.5
**Medication to prevent or relieve post-vaccination side effects**		
Yes	334	56.6
No	173	29.3
Missing	83	14.1
**Severity of experienced side effects**		
Stronger in the first dose than the second dose	98	16.6
Stronger in the second dose than the first dose	238	40.3
In both doses	44	7.5
Stronger in the first dose but I only got one dose because it was Janssen or I had the COVID-19	11	1.9

I did not have effects in any dose	177	30.0
Missing	22	3.7

Table S2: Side effects in the first dose per COVID-19 vaccine (in percentage).

%	Fever<38º	Fever≥38º	Myalgia	Arm soreness, redness, swelling	Nausea	Vomiting	Red, itchy, swollen, or painful rash	Headache	Diarrhea	Loss of appetite	Sweating	Chills	Enlarged lymph nodes	Altered menstrual cycle	Tiredness, sleepiness, dizziness
AstraZeneca/Vaxzevria	21.5	26.7	43.3	35.4	10.7	2.4	1.5	51.3	0.0	8.5	17.6	39.3	2.6	0.0	19.1
Janssen	10.5	5.3	21.1	10.5	0.0	0.0	5.3	21.1	0.0	10.5	15.8	10.5	0.0	0.0	15.8
Moderna	8.0	4.0	17.6	62.4	4.0	1.6	4.8	19.2	3.9	3.2	4.0	8.8	5.6	2.4	9.6
Pfizer	3.7	1.7	9.6	38.3	2.4	0.2	1.7	10.1	1.4	0.7	0.7	6.3	1.6	1.0	5.9
Significant differences	*	*	*	*	*	*	*	*		*	*	*	*	*	*

*Significant results of Pearson Chi-Square

Table S3: Side effects in the second dose per COVID-19 vaccine (in percentage).

%	Fever<38 º	Fever≥38 º	Myalgia	Arm soreness, redness, swelling	Nausea	Vomiting	Red, itchy, swollen, or painful rash	Headache	Diarrhea	Loss of appetite	Sweating	Chills	Enlarged lymph nodes	Altered menstrual cycle	Tiredness, sleepiness, dizziness
AstraZeneca/Vaxzevria	0.0	0.0	16.6	9.1	0.0	0.0	0.0	8.3	0.0	0.0	0.0	0.0	0.0	0.0	0.0
Moderna	26.0	13.0	44.2	57.1	7.8	2.6	3.9	33.8	3.9	6.5	9.1	33.8	5.2	1.3	13.0
Pfizer	12.8	6.2	22.7	30.4	6.2	1.9	0.8	22.5	1.7	2.9	2.5	11.6	3.5	0.6	10.5
Significant differences	*	*	*	*			*	*			*	*			*

*Significant results of Pearson Chi-Square

Table S4: Associations between experience side effects in the first dose of COVID-19 vaccine and weight status (reference: obese). Results from Multinomial Logistic Regression Models.

	Underweight (unadjusted model)		Underweight (adjusted model)		Normal weight (unadjusted model)		Normal weight (adjusted model)		Overweight (unadjusted model)		Overweight (adjusted model)		Obese (ref)	Obese (ref)
	**OR**	**95% CI**	**OR**	**95% CI**	**OR**	**95% CI**	**OR**	**95% CI**	**OR**	**95% CI**	**OR**	**95% CI**	**OR**	**95% CI**

Fever<38º	**3.90**	**1.19-12.75**	1.87	0.58-6.07	2.09	0.94-4.66	1.40	0.64-3.08	1.63	0.70-3.80	1.60	0.70-3.62	Ref.	Ref.
Fever≥38º	**6.76**	**2.24-20.49**	1.57	0.42-5.83	**2.50**	**1.13-5.55**	0.96	0.39-2.46	0.99	0.41-2.38	1.06	0.39-2.90	Ref.	Ref.
Myalgia	**6.64**	**2.62-16.85**	2.20	0.73-6.62	**1.80**	**1.56-5.03**	1.56	0.79-3.10	1.22	0.65-2.31	1.32	0.63-2.74	Ref.	Ref.
Arm soreness, redness, swelling	2.83	1.20-6.64	1.32	0.53-3.24	**1.93**	**1.24-3.02**	1.31	0.81-2.11	1.31	0.82-2.11	1.45	0.88-2.39	Ref.	Ref.
Nausea	0.25	0.02-4.01	0.77	0.03-17.97	0.23	0.03-1.62	0.42	0.05-3.59	0.55	0.06-4.57	0.47	0.05-4.55	Ref.	Ref.
Vomiting	*		*		*		*		*		*		Ref.	Ref.
Red, itchy, swollen, or painful rash	1.00	0.03-29.91	2.74	0.06-127.31	0.54	0.06-4.70	0.96	0.09-10.43	1.00	0.09-11.05	0.87	0.06-11.48	Ref.	Ref.
Headache	**3.88**	**1.55-9.17**	1.14	0.43-3.07	2.13	1.27-3.55	1.14	0.65-198	1.06	0.61-1.85	1.12	0.62-2.00	Ref.	Ref.
Diarrhea	*		*		1.06	0.26-4.76	0.85	0.13-5.70	0.94	0.19-4.74	1.07	0.14-8.06	Ref.	Ref.

Loss of appetite	*		*		*		*		*		*		Ref.	Ref.
Sweating	0.36	0.03-5.18	2.40	0.12-46.90	0.32	0.05-1.92	1.04	0.15-7.40	0.92	0.13-6.52	0.91	0.11-7.33	Ref.	Ref.
Chills	**5.42**	**2.10-13.99**	1.62	0.47-5.59	**2.29**	**1.25-4.20**	1.14	0.53-2.48	1.04	0.58-2.02	1.06	0.47-2.43	Ref.	Ref.
Enlarged lymph nodes	*		*		3.15	0.42-23.84	1.77	0.28-11.21	2.55	0.31-20.63	3.00	0.44-20.24	Ref.	Ref.
Altered menstrual cycle	*		*		*		*		*		*		Ref.	Ref.
Tiredness, sleepiness, dizziness	0.78	0.06-10.40	2.26	0.15-34,78	0.51	0.09-2.27	0.94	0.17-5.32	0.95	0.16-5.64	0.90	0.15-5.39		

Statistically significant results shown in bold font.

Adjusted models were adjusted for age and sex.

* Model was unstable, and analyses could not be run.

Table S5: Associations between experience side effects in the second dose of COVID-19 vaccine and weight status (reference: obese). Results from Multinomial Logistic Regression Models.

	Underweight (unadjusted model)		Underweight (adjusted model)		Normal weight (unadjusted model)		Normal weight (adjusted model)		Overweight (unadjusted model)		Overweight (adjusted model)		Obese (ref)	Obese (ref)
	**OR**	**95% CI**	**OR**	**95% CI**	**OR**	**95% CI**	**OR**	**95% CI**	**OR**	**95% CI**	**OR**	**95% CI**	**OR**	**95% CI**
Fever<38º	1.17	0.12-11.23	1.00	0.09-11.98	1.47	0.60-3.62	1.41	0.52-3.80	1.24	0.48-3.21	1.24	0.44-3.52	Ref.	Ref.
Fever≥38º	*		*		0.87	0.19-4.09	0.85	0.25-2.91	0.60	0.11-3.35	0.52	0.13-2.01	Ref.	Ref.
Myalgia	**6.64**	**2.62-16.85**	2.20	0.73-6.62	**1.80**	**1.56-5.03**	1.56	0.79-3.10	1.22	0.65-2.31	1.32	0.63-2.74	Ref.	Ref.
Arm soreness, redness, swelling	**2.83**	**1.20-6.64**	1.32	0.53-3.24	**1.93**	**1.24-3.02**	1.31	0.81-2.11	1.31	0.82-2.11	1.45	0.88-2.39	Ref.	Ref.
Nausea	0.25	0.02-4.01	0.77	0.03-17.97	0.23	0.03-1.62	0.42	0.05-3.59	0.55	0.06-4.57	0.47	0.05-4.55	Ref.	Ref.
Vomiting	*		*		*		*		*		*		Ref.	Ref.

Red, itchy, swollen, or painful rash	1.00	0.03-29.91	2.74	0.06-127.31	0.54	0.06-4.70	0.96	0.09-10.43	1.00	0.09-11.05	0.87	0.06-11.48	Ref.	Ref.
Headache	**3.88**	**1.55-9.17**	1.14	0.43-3.07	2.13	1.27-3.55	1.14	0.65-198	1.06	0.61-1.85	1.12	0.62-2.00	Ref.	Ref.
Diarrhea	*		*		1.06	0.26-4.76	0.85	0.13-5.70	0.94	0.19-4.74	1.07	0.14-8.06	Ref.	Ref.
Loss of appetite	*		*		*		*		*		*		Ref.	Ref.
Sweating	0.36	0.03-5.18	2.40	0.12-46.90	0.32	0.05-1.92	1.04	0.15-7.40	0.92	0.13-6.52	0.91	0.11-7.33	Ref.	Ref.
Chills	**5.42**	**2.10-13.99**	1.62	0.47-5.59	**2.29**	**1.25-4.20**	1.14	0.53-2.48	1.04	0.58-2.02	1.06	0.47-2.43	Ref.	Ref.
Enlarged lymph nodes	*		*		3.15	0.42-23.84	1.77	0.28-11.21	2.55	0.31-20.63	3.00	0.44-20.24	Ref.	Ref.
Altered menstrual cycle	*		*		*		*		*		*		Ref.	Ref.

Tiredness, sleepiness, dizziness	0.78	0.06-10.40	2.26	0.15-34,78	0.51	0.09-2.27	0.94	0.17-5.32	0.95	0.16-5.64	0.90	0.15-5.39		

Statistically significant results shown in bold font.

Adjusted models were adjusted for age and sex.

* Model was unstable, and analyses could not be run.

Table S6: Associations between weight status (reference: overweight/obese) and side effects experienced in the first dose of COVID-19 vaccine by administered vaccine (Pfizer vs AstraZeneca/Vaxzevria).

Results from Multinomial Logistic Regression Models.

	First dose of **Pfizer:** Underweight/Normal weight		First dose of **AstraZeneca/Vaxzevria** Underweight/Normal weight	
	**OR**	**95% CI**	**OR**	**95% CI**
Fever<38º	0.40	0.11-1.50	1.06	0.71-1.0
Fever≥38º	0.95	0.53-1.71	0.98	0.60-1.62
Myalgia	1.02	0.51-2.03	1.45	0.95-2.30

Arm soreness, redness, swelling	0.94	0.60-1.46	0.97	0.66-1.43
Nausea	1.39	0.42-3.90	1.74	0.92-3.31
Vomiting	-	-	7.54	0.95-59.57
Red, itchy, swollen, or painful rash	1.11	0.36-3.42	0.50	0.16-1.51
Headache	0.78	0.42-1.42	1.23	0.82-1.84
Diarrhea	0.97	0.25-3.78	0.67	0.25-1.78
Loss of appetite	0.84	0.18-3.99	1.36	0.49-3.73
Sweating	0.24	0.05-1.13	1.03	0.57-1.89
Chills	0.68	0.29-1.59	1.20	0.78-1.84
Enlarged lymph nodes	0.95	0.36-2.51	1.38	0.54-3.51
Altered menstrual cycle	0.73	0.39-1.35	**0.15**	**0.07-0.33**

Tiredness, sleepiness, dizziness	0.75	0.34-1.68	0.97	0.53-1.75

Statistically significant results shown in bold font.

All these models were adjusted for age, sex, education status and medication to prevent or relieve post-vaccination side effects.
